# Exercise Capacity Is Independent of Respiratory Muscle Strength in Patients with Chronic Heart Failure

**DOI:** 10.3390/jcm11133875

**Published:** 2022-07-04

**Authors:** Ali Albarrati, Abdulrahman Aseeri, Mohammed Taher, Monira I. Aldhahi, Rakan I. Nazer

**Affiliations:** 1 Rehabilitation Sciences Department, College of Applied Medical Sciences, King Saud University, Riyadh 11451, Saudi Arabia; momarar@ksu.edu.sa; 2 King Salman Hospital, Riyadh 12769, Saudi Arabia; physiotherapist1981@yahoo.com; 3 Department of Rehabilitation Sciences, College of Health and Rehabilitation Sciences, Princess Nourah bint Abdulrahman University, Riyadh 11671, Saudi Arabia; mialdhahi@pnu.edu.sa; 4 Cardiac Sciences Department, College of Medicine, King Saud University, Riyadh 11451, Saudi Arabia; raknazer@ksu.edu.sa

**Keywords:** breathlessness, dyspnea, exercise intolerance, respiratory muscle strength

## Abstract

Background: Exercise intolerance in patients with chronic heart failure (CHF) is associated with a number of factors, including breathlessness and respiratory muscle weakness. However, many studies reported controversial results, and as yet there is no study on Arabic patients with CHF. This study aimed to examine the impact of breathlessness and respiratory muscle strength on exercise capacity in Arabic patients with CHF. Methods: This was a cross-sectional study, involving 42 stable adult male patients with CHF with a reduced ejection fraction and 42 controls who were free from cardiorespiratory and neuromuscular diseases. Patients with CHF and the controls underwent respiratory muscle strength tests and a six-minute walk test (6MWT), and the measurements were taken. Dyspnea was recorded using the modified Medical Research Council (mMRC) scale, along with the number of comorbidities. Results: Patients with CHF and controls were similar in age and sex. Patients with CHF had a greater number of comorbidities, a higher dyspnea score, a lower 6MWT score, and lower respiratory muscle strength (*p* < 0.001). Only 7% of patients with CHF had weak inspiratory muscle strength (<60% of that predicted) and 40% terminated the 6MWT due to dyspnea. The 6MWT was associated with mMRC *(rs* = −0.548, *p* < 0.001) but not with respiratory muscle strength (*p* > 0.05). Conclusions: Exercise intolerance in patients with CHF was associated with dyspnea and was independent of respiratory muscle strength.

## 1. Introduction

Chronic heart failure (CHF) is a multifaceted disease that is associated with several manifestations beyond the heart, including exercise intolerance, breathlessness, and reduced respiratory muscle strength. Exercise intolerance is the hallmark symptom of CHF, and patients with CHF experience a progressive decline in exercise capacity, yet cardiac physiological indices are not severely impaired [1,2].

Exercise capacity is an important predictor of readmissions, morbidity, and mortality among patients with CHF. The mechanism of exercise intolerance is yet to be revealed; however, a number of contributing factors play a role, such as breathlessness and reduced respiratory muscle strength.

Breathlessness is a term describing a patient’s experience and sensations, which is usually different from the biomedical term of dyspnea [3,4]. The perception of breathlessness is specifically linked to respiratory muscle strength, whereby weak respiratory muscles in patients with CHF significantly contribute to dyspnea during daily activity [5]. Dyspnea negatively impacts individuals’ exercise capacity, and it is a better prognosticator of mortality than angina among patients with cardiac disease. Resting dyspnea usually occurs after patients with CHF begin to suffer from exertional dyspnea; therefore, it is commonly used to assess clinical functioning among patients with CHF [5]. However, the exact mechanism of dyspnea needs to be elucidated.

Several studies have examined the link between respiratory muscle strength and dyspnea and their effect on exercise intolerance among patients with CHF [6,7,8,9,10]. However, these studies have yielded controversial findings, due to the heterogeneity of their samples and their methods of exercise capacity and respiratory muscle strength assessment. Furthermore, other researchers have taken this a step further and have assessed if exercise intolerance could be improved by training the respiratory muscles [11,12,13,14,15]. Unfortunately, the results of these studies are contentious, as the patients with CHF recruited for these studies were heterogeneous and presented with other comorbidities that had an impact on exercise capacity. Therefore, there is a need for a study to control for the most controversial variables among previous studies, which have led elsewhere to inconsistencies between the findings. This study aimed to examine the factors associated with exercise intolerance in stable patients with CHF.

## 2. Materials and Methods

### 2.1. Setting and Participants

This was a cross-sectional study involving stable adult male patients with CHF who presented with reduced ejection fraction. Patients with CHF who were suitable for the study were identified via the King Salman Heart Center’s outpatient clinic. Patients with CHF were included in the study if they were clinically stable for more than 3 months and had not received any adjustment therapy one month prior to recruitment. Patients were excluded if they had unstable angina, myocardial infarction within the previous month, cardiac surgery in the past three months, chronic respiratory disease, pulmonary artery hypertension, chronic metabolic disorders, severe musculoskeletal or neurological disorders, or infectious disease. Patients were also excluded if they were treated with steroids, hormones, or cancer chemotherapy, as this can affect or alter the patient’s central and peripheral muscle strength and exercise performance. Controls without CHF were recruited from community centers, the patients’ relatives, and smoking cessation clinic databases. Controls were included if they had no cardiorespiratory disease and similar exclusion criteria were applied to the patients with CHF. 

### 2.2. Ethical Approval

The study was approved by the King Saud University ethics committee (IRB number: E-19-4472) and the King Fahad Medical City ethics committee (IRB number: 20-104E); participants signed a consent form before enrolment. The study was conducted during the period from March 2020 to October 2020 and was performed in accordance with the principles of the Declaration of Helsinki.

### 2.3. Sample Size Estimation

Sample size estimation was calculated using the G*power program, version 3.0.10 [16], based on an estimated minimum correlation of 0.30 between resting breathlessness and exercise intolerance, with an alpha level of 0.05 and a power of 0.80. The required sample size was found to be 84 for both groups.

### 2.4. Procedures

#### 2.4.1. Demographic and Clinical Data Assessment

All participants had their age, height, and weight details taken, and their body mass index (BMI) was calculated and recorded in kg/m^2^. All patients with CHF were interviewed to record their medical history and medications and had their vital signs taken using a Welch Allyn 52000 monitor (IL, USA). The left ventricle ejection fraction (LVEF) value over four weeks was recorded from the patient’s electronic file.

#### 2.4.2. Respiratory Muscle Strength Testing

All participants underwent respiratory muscle strength measurements, using a reliable and valid device (MicroRPM CareFusion 232 (UK) Ltd., UK) [17,18]. All tests were performed according to the American Thoracic Association (ATS) guidelines [19].

Respiratory muscle strength was assessed while the participant was sitting on a chair with their feet flat on the floor and their back against the back of the chair, with their hands resting on their thighs and their shoulders back. The procedures were thoroughly explained to the participants, and they were given time to practice before the test. During these tests, the participants were encouraged verbally to perform to the best of their ability.

##### Maximum Inspiratory Pressure (MIP)

The participant was instructed to insert the mouthpiece into the mouth, ensuring that the flange was positioned over the gums and inside the lips. While the ‘bite block’ was held between the teeth, the participant was asked to exhale to their residual volume (RV), lungs empty, then to perform a ‘Mueller’ maneuver, a forced inhalation using as much effort as possible for as long as possible, for a minimum of 2 s. Each participant repeated the test three times, with a resting period of 30 s between each attempt, and the highest value was recorded.

##### Maximum Expiratory Pressure (MEP)

The participant was instructed to insert the mouthpiece into the mouth, ensuring that the flange was positioned over the gums and inside the lips, while the ‘bite blocks’ were held between the teeth, then the participant was asked to inhale to their total lung capacity (TLC), lungs full, then to perform a ‘Valsalva’ maneuver, a forced exhalation using as much effort as possible for as long as possible, for a minimum 2 s. The test was repeated three times, with a resting period of 30 s between each attempt, and the highest value was recorded.

##### Sniff Nasal Inspiratory Pressure (SNIP)

After choosing and placing an appropriate nostril probe inside the participant’s nostril, the probe was connected to the MicroRPM device. The participant was then asked to maintain normal breathing, with the mouth closed. At the end of a relaxed expiration, showing the functional residual capacity (FRC), the participant was asked to take a sharp and quick inhalation of maximum inspiratory effort. The test was repeated at least six times, with a resting period of 10 s between each attempt, and the highest value was recorded. Heritier et al. [18] described a close relationship between the Sniff Pnasal and Sniff Poes (r = 0.99) values in normal subjects without nasal obstruction.

#### 2.4.3. Six-Minute Walk Test (6MWT)

The 6MWT was carried out in accordance with a protocol adapted from the ATS guidelines, using a 30-m straight-level course in an enclosed corridor [20]. The distance covered was recorded in meters. The participants were asked to rate their breathlessness before and immediately after the test, using the Modified Borg Scale, which is reliable and is valid in assessing the level of breathlessness in cardiac patients [21]. A score of 0 would indicate nothing at all; 0.5 would indicate very, very slight breathlessness, followed by the whole numbers of 1 through to 10.

#### 2.4.4. Modified Medical Research Council (mMRC) Scale

Dyspnea in daily life was evaluated using the modifiable Medical Research Council (mMRC scale) [22]. The participants were asked to choose a grade (number) from 0 to 4 that best described their level of breathlessness during the day, in which 0 meant no breathlessness at all at rest, to 4, which is too breathless when at rest.

### 2.5. Data Analysis

Data analysis was performed using IBM SPSS Statistics for Windows, Version 27.0 (IBM Corp., Armonk, NY, USA). Data normality was examined prior to data analysis. For descriptive statistics, parametric data were presented as a mean and SD, and nonparametric data as the median and interquartile range (IQR). The independent *t*-test was used to compare the groups for parametric data, and the Mann–Whitney U-test was used for nonparametric variable comparisons. An analysis of covariance (ANCOVA) was used to examine the differences between subgroups, after controlling for possible confounding factors. The correlation between variables was examined using a Spearman correlation for nonparametric data or a Pearson correlation for parametric data in each group. Multiple linear regression was conducted to determine the predictive factors of exercise capacity in the patient group. The MIP reference values were calculated using the Evans and Whitelaw algorithm [23]: male MIP 120 − (0.41 × age), and male MEP = 174 − (0.83 × age). Patients with CHF were divided into those with either normal inspiratory muscle strength or inspiratory muscle weakness if they scored below 60% of the predicted value [23]. Patients with CHF were also divided into dyspneic, for those who terminated the 6MWT prematurely, and non-dyspneic patients, for those who completed the 6MWT.

## 3. Results

### 3.1. Participants’ Demographic and Clinical Characteristics

Forty-two patients with CHF, showing a reduced ejection fraction, and forty-two controls were enrolled in the current study. The clinical and physical characteristics of the participants are presented in Table 1. Patients with CHF and the controls were similar in age, BMI, resting HR, resting BP, and resting O_2_ saturation, but patients with CHF had greater mMRC score and a greater number of comorbidities, compared with controls (Table 1). The origin of the patients’ CHF was related to ischemic heart disease in 22 (52%) of the patients, while the remaining 20 (48%) had cardiomyopathy.

Patients with CHF had lower 6MWT values and respiratory muscle strength (MIP, MEP, and SNIP) compared with controls, even after adjusting for the mMRC score and the number of comorbidities (*p* < 0.05) (Table 1).

### 3.2. Correlation between Exercise Capacity, Dyspnea, and Ejection Fraction

The 6MWT was inversely correlated with mMRC *(rs* = −0.548, *p* < 0.001) in patients with CHF, but this was not evident in the controls. There was no correlation between 6MWT and the number of comorbidities, neither in patients with CHF (*rs* = −0.297, *p* > 0.05), nor in controls (*rs* = −0.385, *p* > 0.05). Neither exercise capacity nor resting or exertional dyspnea correlated with the left ventricle function ejection fraction (*p* > 0.05). The relationship between the 6MWT and mMRC remained significant, even after adjusting for the number of comorbidities and the left ventricle function ejection fraction (adjusted R^2^ = 0.49, F = 31.137, *p* < 0.001). 

### 3.3. Correlation between Exercise Capacity and Respiratory Muscle Strength

In patients with CHF, neither inspiratory muscle strength, MIP (*rs* = 0.26), or SNIP (*rs* = 0.103), nor expiratory muscle strength or MEP (*rs* = 0.04) correlated with exercise capacity in the 6MWT. This finding was similar to those in the controls. The number of patients with CHF who terminated the 6MWT due to breathlessness was 17 (40%). Dyspneic patients with CHF who terminated the 6MWT prematurely had a lower exercise capacity compared to those non-dyspneic patients with CHF who did complete the test (Table 2). Both groups had similar ages, LVEF, and the number of comorbidities. There was no difference in respiratory muscle strength between the dyspneic and non-dyspneic groups (Table 2).

### 3.4. Correlation between Respiratory Muscle Strength and Dyspnea

Only three patients had inspiratory muscle weakness, with an MIP < 60% predicted, and six patients had expiratory muscle weakness, with a, MEP < 80% predicted. In patients with CHF, there were no correlations between mMRC and inspiratory muscle strength, MIP (*rs* = −0.252, *p* > 0.05), SNIP (*rs* = −0.152, *p* > 0.05) or expiratory muscle strength, MEP (*rs* = −0.142, *p* > 0.05). This did not change, even after controlling for the number of comorbidities and LVEF (F = 0.649, *p* = 0.425). Similarly, the control group showed no correlation between respiratory muscle strength and mMRC (*p* > 0.05). Dyspneic patients with CHF showed no significant differences in inspiratory and expiratory muscle strength, compared to non-dyspneic patients with CHF (Table 2).

### 3.5. Predictive Factors for Exercise Capacity

In the patients with CHF, stepwise multivariate regression analysis, after controlling for age, LVEF, and the number of comorbidities, showed that mMRC explained 38% of the variability in the 6MWT (adjusted R^2^ = 0.38, F = 26.15, *p* < 0.001).

## 4. Discussion

This is the first study examining the factors associated with exercise intolerance among Arab patients with CHF, with reduced ejection fraction, and comparing them with age-matched controls. The study found that patients with CHF had lower exercise capacity, higher levels of dyspnea, and lower respiratory muscle strength compared with the age-matched controls. The results showed a negative correlation between exercise capacity and dyspnea in patients with CHF, but there was no correlation between dyspnea and respiratory muscle strength in patients with CHF. Both groups showed no correlation between exercise capacity and respiratory muscle strength.

Reduced exercise capacity in patients with CHF is multifactorial [24]. In this study, we found that reduced exercise capacity in patients with CHF was associated with an increased level of dyspnea, independent of the number of comorbidities and the ejection fraction of the left ventricle. An increased level of dyspnea during exercise is the hallmark of exercise intolerance in patients with CHF [25,26]. Dyspnea is multidimensional and derives from interactions between several domains, including physiological, psychological, social, and environmental factors [27]. This complex interaction triggers the sensation of dyspnea, and patients with CHF experience it differently from the way they perceive it. Dyspnea is mistakenly classified by some clinicians as a clinical sign in patients with CHF when the patient has an increased respiratory rate or has difficulty breathing. Unlike dyspnea, breathlessness is a common clinical symptom in CHF and is multifactorial [24]. It reflects the patient’s views and experiences and has an impact on daily living activities. Unlike other studies on CHF, in this study, we recruited stable patients with CHF who were free from respiratory comorbidities, to eliminate the respiratory system as a source of breathlessness or as a contributing factor to increased breathlessness and reduced exercise capacity. Additionally, we assessed the different domains of dyspnea, including sensory-perceptual experience (i.e., the patient’s breathing sensations), via a modified Borg scale, and the impact of the symptom on functional ability via a modified MRC. We further examined the association between breathlessness and exercise capacity between patients with CHF who terminated the test prematurely, due to dyspnea, and those who completed the test. Dyspnea was the limiting factor in 40% of patients with CHF in our study. This finding is in agreement with a study by Mancini et al. [8], who reported that 20% of studied patients with CHF were unable to complete exercise testing, due to dyspnea. Similarly, Clark et al. [8] found that 160 out of 222 patients with CHF terminated the exercise testing because of dyspnea. This implies that breathlessness in those patients is probably triggered by their perception, which conveys an abnormal or faulty response from the brain.

In our study, neither exercise capacity nor resting dyspnea or exertional dyspnea was associated with left ventricle ejection fraction. Several studies in patients with CHF demonstrated that the baseline left ventricular ejection fraction is poorly related to exercise capacity and baseline dyspnea [28,29]. Witte et al. [28] and Hacker et al. [29] reported that patients with CHF terminated exercise testing because of dyspnea, irrespective of their left ventricle function, ventilatory effects, or peak oxygen consumption.

In the current study, patients with CHF had lower inspiratory and expiratory muscle strength compared with the controls. This could be attributed to an insufficient oxygen supply to the respiratory muscles, the depletion of heavy-chain myosin, and alterations in the respiratory muscle fibers [24]. However, whether these changes in respiratory muscles are contributing to exercise intolerance and increased breathlessness remains contentious. We found that lower respiratory muscle strength was not associated with reduced exercise capacity. A study by Mancini et al. [8] did not find a reduction in diaphragm strength during phrenic nerve stimulation. Similarly, as in our study, Evans and colleagues reported that there was no correlation between respiratory muscle strength and exercise capacity [6].

The reduced strength of inspiratory or expiratory muscles does not imply that these muscles are weak or are not functioning well compared to the predicted values or individuals without CHF. Previous, randomized controlled trials and cross-sectional studies proposed that patients with CHF have respiratory muscle weakness, contributing to exercise tolerance [10,11,12,13,14,15]. Nevertheless, these studies failed to highlight whether the reduced respiratory muscle strength in their studies is clinically meaningful and will significantly alter the muscles’ strength. Additionally, these studies have included patients with chronic kidney disease (CKD) and chronic obstructive pulmonary disease (COPD), which are well-known comorbidities that induce musculoskeletal maladaptation [30,31]. Chronic diseases, such as CKD and COPD, are associated with increased systemic inflammation, malnutrition, and musculoskeletal changes, which are well-established risk factors for musculoskeletal weakness and exercise intolerance, independently of CHF [32].

This study demonstrated that neither resting dyspnea nor exertional dyspnea was associated with reduced respiratory muscle strength. We further showed that respiratory muscle strength did not differ between dyspneic patients who terminated the 6MWT prematurely and non-dyspneic patients. This is further supported by the absence of oxygen desaturation in the peripheral musculoskeletal tissues, which may eliminate the lungs as a primary source of dyspnea. In line with this finding, Mancini and colleagues [8] found no association between peak oxygen consumption and dyspnea in patients with CHF. 

Exercise-based cardiac rehabilitation is an essential element in the management of patients with CHF. Considering the results of this study, the cardiac rehabilitation team, including cardiologists, specialists in physical and rehabilitation medicine, and physical therapists need to provide individualized cardiac rehabilitation programs according to each patient’s physical and clinical findings [33,34]. Dyspnea in patients with CHF who are free from respiratory disease is a source of exercise intolerance that extends beyond the cardiorespiratory system [24,25,26,32]. Tackling dyspnea to improve exercise capacity via pharmacological and non-pharmacological (i.e., psychological) interventions should be considered [33,34].

### Limitations

The current study should nevertheless not be interpreted without taking several factors into consideration. First, all patients with CHF recruited in this study were males; therefore, these results cannot be extrapolated to females with CHF. Patients with a preserved LVEF were not included in the study. The prevalence of CHF is more common in males than in females, and males are more susceptible to the risk factors associated with CHF. A second limitation of the study was that even though some patients had very low 6MWT and LVEF values, over a quarter (28%) of the patients who were recruited in this study were in stage III_IV of the NYHA Functional Classification. Although the NYHA Functional Classification is frequently used among cardiologists in cases of CHF, it is a subjective and weak outcome measure by which to discriminate between functional impairment in patients with CHF. Finally, we did not examine the effects of medications on the reported outcome measures. However, heart failure medications (i.e., vasodilators and inotropes) have minimal effects on exercise capacity and exertional dyspnea.

## 5. Conclusions

This study found that exercise intolerance in patients with CHF was related to a higher level of dyspnea, independent of respiratory muscle strength. The level of dyspnea was not associated with respiratory muscle strength, although the patients with CHF had lower respiratory muscle strength but were not weak compared with the controls.

## Figures and Tables

**Table 1:** Physical and clinical characteristics of participants.

Variable	Patients with CHF (*N* = 42)	Controls (*N* = 42)	*p*-Value
Age (years)	53 (14)	52 (13)	0.802
Weight (kg)	84.44 (15.90)	87.00 (16.23)	0.951
BM (kg/m^2^)	30.1 (0.88)	29.6 (1)	0.761
LVEFF (%)	29.42 (9.77)	-	-
Resting HR (beats/min)	74 (16)	79 (9)	0.103
Resting O_2_ saturation (%)	98 (2)	97 (1)	0.824
Resting Systolic BP (mmHg)	127 (22)	124(16)	0.086
Resting Diastolic BP (mmHg)	72 (13)	84 (9)	0.077
MIP (cmH_2_O)	82 (23)	113 (20)	<0.001
MEP (cmH_2_O)	114 (24)	149 (26)	<0.001
SNIP (cmH_2_O)	85 (17)	109 (17)	<0.001
6MWT (m)	404 (80)	520 (68)	<0.001
mMRC	1 (0–3)	0	<0.001
Number of comorbidities	2 (1–3)	0 (0–0.25)	0.001
Borg Scale	1 (1–1)	0 (0)	<0.01
NYHA	I = 11		
	II = 21		
	III = 7		
	IV = 3		

Data are presented as mean (SD), median and interquartile range, or the number and percentage. Abbreviations: BP: blood pressure; LVEF: left ventricle ejection fraction; MIP: maximum inspiratory pressure; MEP: maximum expiratory pressure; mMRC: modified Medical Research Council scale; SNIP: sniff nasal inspiratory pressure; 6MWT: six-minute walking test; NYHA: New York Heart Association.

**Table 2:** Clinical and physiological characteristics of dyspneic and non-dyspneic groups.

	Dyspneic Group (*N* = 17)	Non- Dyspneic Group (*N* = 25)	*p*-Value
Age (years)	56 (14)	51 (14)	0.255
LVEF (%)	31 (11)	28 (9)	0.519
MIP (cmH_2_O)	79 (16)	87 (22)	0.254
SNIP (cmH_2_O)	81(18)	85 (19)	0.656
MEP (cmH_2_O)	114 (26)	114 (23)	0.950
6MWT (m)	334 (70)	456 (35)	0.001
mMRC	1.3 (0.6)	0.7(0.6)	0.005
* Number of comorbidities	2.3 (0.95)	2 (1)	0.165
* Borg_ Pre-6MWT	1 (0.4)	1 (1)	0.870
* Borg_ Post-6MWT	4 (2)	2 (1)	0.187

Data are presented as mean (SD) unless otherwise indicated. * Geometric mean. Abbreviations: MIP: maximum inspiratory pressure; MEP: maximum expiratory pressure; mMRC: modified Medical Research Council scale; LVEF: left ventricle ejection fraction; SNIP: sniff nasal inspiratory pressure; 6MWT: six-minute walking test.

## Data Availability

The datasets used and/or analyzed during the current study are available upon reasonable request.

## References

[1] Poole D.C., Richardson R.S., Haykowsky M.J., Hirai D.M., Musch T.I. (2018). Exercise limitations in heart failure with reduced and preserved ejection fraction. J. Appl. Physiol..

[2] Piña I.L., Apstein C.S., Balady G.J., Belardinelli R., Chaitman B.R., Duscha B.D., Fletcher B.J., Fleg J.L., Myers J.N., Sullivan M.J. (2003). Exercise and heart failure: A statement from the American Heart Association Committee on exercise, rehabilitation, and prevention. Circulation.

[3] Parshall M.B., Schwartzstein R.M., Adams L., Banzett R.B., Manning H.L., Bourbeau J., Calverley P.M., Gift A.G., Harver A., Lareau S.C. (2012). An Official American Thoracic Society Statement: Update on the Mechanisms, Assessment, and Management of Dyspnea. Am. J. Respir. Crit. Care Med..

[4] Lavie C.J., Shah S.B., Mehra M.R. (2019). The Dilemma of Exertional Dyspnea and Diagnosis of Heart Failure: Convergent and Discriminant Validity. JACC Cardiovasc. Imaging.

[5] McParland C., Krishnan B., Wang Y., Gallagher C.G. (1992). Inspiratory Muscle Weakness and Dyspnea in Chronic Heart Failure. Am. Rev. Respir. Dis..

[6] A Evans S., Watson L., Hawkins M., Cowley A.J., Johnston I.D., Kinnear W.J. (1995). Respiratory muscle strength in chronic heart failure. Thorax.

[7] Hamazaki N., Kamiya K., Matsuzawa R., Nozaki K., Ichikawa T., Tanaka S., Nakamura T., Yamashita M., Maekawa E., Noda C. (2019). Prevalence and prognosis of respiratory muscle weakness in heart failure patients with preserved ejection fraction. Respir. Med..

[8] Mancini D.M., Henson D., LaManca J., Levine S. (1992). Respiratory muscle function and dyspnea in patients with chronic congestive heart failure. Circulation.

[9] Meyer F.J., Borst M.M., Zugck C., Kirschke A., Schellberg D., Kübler W., Haass M. (2001). Respiratory muscle dysfunction in congestive heart failure: Clinical correlation and prognostic significance. Circulation.

[10] Nakagawa N.K., Diz M.A., Kawauchi T.S., De Andrade G.N., Umeda I.I.K., Murakami F.M., Oliveira-Maul J.P., Nascimento J.A., Nunes N., Takada J.Y. (2019). Risk Factors for Inspiratory Muscle Weakness in Chronic Heart Failure. Respir. Care.

[11] Nishimura Y., Maeda H., Tanaka K., Nakamura H., Hashimoto Y., Yokohama M. (1994). Respiratory Muscle Strength and Hemodynamics in Chronic Heart Failure. Chest.

[12] Hamilton D.M., Haennel R.G. (2000). Validity and Reliability of the 6-Minute Walk Test in a Cardiac Rehabilitation Population. J. Cardiopulm. Rehabil..

[13] Laoutaris I.D., Dritsas A., Adamopoulos S., Brown M.D., Cokkinos D.V. (2008). Effects of Inspiratory Muscle Training in Patients With Chronic Heart Failure. J. Am. Coll. Cardiol..

[14] Bosnak-Guclu M., Arikan H., Savci S., Inal-Ince D., Tulumen E., Aytemir K., Tokgözoglu L. (2011). Effects of inspiratory muscle training in patients with heart failure. Respir. Med..

[15] Kasahara Y., Izawa K.P., Watanabe S., Osada N., Omiya K. (2015). The relation of respiratory muscle strength to disease severity and abnormal ventilation during exercise in chronic heart failure patients. Res. Cardiovasc. Med..

[16] Faul F., Erdfelder E., Buchner A., Lang A.-G. (2009). Statistical power analyses using G*Power 3.1: Tests for correlation and regression analyses. Behav. Res. Methods.

[17] Dimitriadis Z., Kapreli E., Konstantinidou I., Oldham J., Strimpakos N. (2011). Test/Retest Reliability of Maximum Mouth Pressure Measurements With the MicroRPM in Healthy Volunteers. Respir. Care.

[18] Héritier F., Rahm F., Pasche P., Fitting J.W. (1994). Sniff nasal inspiratory pressure. A noninvasive assessment of inspiratory muscle strength. Am. J. Respir. Crit. Care Med..

[19] Laveneziana P., Albuquerque A., Aliverti A., Babb T., Barreiro E., Dres M., Dubé B.-P., Fauroux B., Gea J., Guenette J.A. (2019). ERS statement on respiratory muscle testing at rest and during exercise. Eur. Respir. J..

[20] (2002). ATS Committee on Proficiency Standards for Clinical Pulmonary Function Laboratories. ATS statement: Guidelines for the six-minute walk test. Am. J. Respir. Crit. Care Med..

[21] Whaley M.H., Brubaker P.H., Kaminsky L.A., Miller C.R. (1997). Validity of Rating of Perceived Exertion During Graded Exercise Testing in Apparently Healthy Adults and Cardiac Patients. J. Cardiopulm. Rehabil..

[22] Munari A.B., A Gulart A., Dos Santos K., Venâncio R.S., Karloh M., Mayer A.F. (2017). Modified Medical Research Council Dyspnea Scale in GOLD Classification Better Reflects Physical Activities of Daily Living. Respir. Care.

[23] Evans J.A., Whitelaw W.A. (2009). The assessment of maximal respiratory mouth pressures in adults. Respir. Care.

[24] Dubé B.-P., Agostoni P., Laveneziana P. (2016). Exertional dyspnoea in chronic heart failure: The role of the lung and respiratory mechanical factors. Eur. Respir. Rev..

[25] Clark A.L., Sparrow J.L., Coats A.J. (1995). Muscle fatigue and dyspnoea in chronic heart failure: Two sides of the same coin?. Eur. Heart J..

[26] Laveneziana P., Agostoni P. (2016). Exertional dyspnoea in cardiorespiratory disorders: The clinical use of cardiopulmonary exercise testing. Eur. Respir. Rev..

[27] Sue D.Y. (2000). Exertional dyspnea in congestive heart failure. Living longer and doing more?. Chest.

[28] A Witte K.K., Nikitin N.P., De Silva R., Cleland J.G.F., Clark A.L. (2004). Exercise capacity and cardiac function assessed by tissue Doppler imaging in chronic heart failure. Heart.

[29] Hacker M., Störk S., Stratakis D., Angermann C.E., Huber R., Hahn K., Tausig A. (2003). Relationship between right ventricular ejection fraction and maximum exercise oxygen consumption: A methodological study in chronic heart failure patients. J. Nucl. Cardiol..

[30] Shiba N., Shimokawa H. (2011). Chronic kidney disease and heart failure—Bidirectional close link and common therapeutic goal. J. Cardiol..

[31] Gea J., Pascual S., Casadevall C., Orozco-Levi M., Barreiro E. (2015). Muscle dysfunction in chronic obstructive pulmonary disease: Update on causes and biological findings. J. Thorac. Dis..

[32] Guazzi M., Myers J., Vicenzi M., Bensimhon D., Chase P., Pinkstaff S., Arena R. (2010). Cardiopulmonary exercise testing characteristics in heart failure patients with and without concomitant chronic obstructive pulmonary disease. Am. Heart J..

[33] Heidenreich P.A., Bozkurt B., Aguilar D., Allen L.A., Byun J.J., Colvin M.M., Deswal A., Drazner M.H., Dunlay S.M., Evers L.R. (2022). 2022 AHA/ACC/HFSA Guideline for the Management of Heart Failure: Executive Summary: A Report of the American College of Cardiology/American Heart Association Joint Committee on Clinical Practice Guidelines. Circulation.

[34] Birke H., Foxvig I., Burns K., Toft U., Hansen A.B.G., Hauge P.I., Foghmar S., Mindegaard R.B., Jakobsen L.M. (2022). Heart Rehabilitation for All (HeRTA): Protocol for a feasibility study and pilot randomized trial. PLoS ONE.

